# Computational strategies for the preconditioned conjugate gradient method applied to ssSNPBLUP, with an application to a multivariate maternal model

**DOI:** 10.1186/s12711-020-00543-9

**Published:** 2020-05-13

**Authors:** Jeremie Vandenplas, Herwin Eding, Maarten Bosmans, Mario P. L. Calus

**Affiliations:** 1 Animal Breeding and Genomics, Wageningen UR, P.O. 338, 6700 AH Wageningen, The Netherlands; 2 CRV BV, Wassenaarweg, 20, 6843 NW Arnhem, The Netherlands; 3 VORtech Scientific Software Engineers, Westlandseweg 40d, 2624 AD Delft, The Netherlands

## Abstract

**Background:**

The single-step single nucleotide polymorphism best linear unbiased prediction (ssSNPBLUP) is one of the single-step evaluations that enable a simultaneous analysis of phenotypic and pedigree information of genotyped and non-genotyped animals with a large number of genotypes. The aim of this study was to develop and illustrate several computational strategies to efficiently solve different ssSNPBLUP systems with a large number of genotypes on current computers.

**Results:**

The different developed strategies were based on simplified computations of some terms of the preconditioner, and on splitting the coefficient matrix of the different ssSNPBLUP systems into multiple parts to perform its multiplication by a vector more efficiently. Some matrices were computed explicitly and stored in memory (e.g. the inverse of the pedigree relationship matrix), or were stored using a compressed form (e.g. the Plink 1 binary form for the genotype matrix), to permit the use of efficient parallel procedures while limiting the required amount of memory. The developed strategies were tested on a bivariate genetic evaluation for livability of calves for the Netherlands and the Flemish region in Belgium. There were 29,885,286 animals in the pedigree, 25,184,654 calf records, and 131,189 genotyped animals. The ssSNPBLUP system required around 18 GB Random Access Memory and 12 h to be solved with the most performing implementation.

**Conclusions:**

Based on our proposed approaches and results, we showed that ssSNPBLUP provides a feasible approach in terms of memory and time requirements to estimate genomic breeding values using current computers.

## Background

Genomic data for livestock often include around 50 thousand single nucleotide polymorphism (SNPs), and are used in genomic prediction to obtain genomic estimated breeding values [1]. While some challenges must be still solved, the method of choice for genomic prediction is currently the so-called single-step genomic best linear unbiased prediction (ssGBLUP) that simultaneously analyses phenotypic and pedigree information of genotyped and non-genotyped animals with genomic information of genotyped animals [1]. ssGBLUP considers genomic information by combining genomic and pedigree relationships into a combined genomic-pedigree relationship matrix [2, 3]. A drawback of ssGBLUP is that it requires the inverse of the genomic relationship matrix (${\mathbf {G}}$), which can be computed up to approximately 100,000 genotyped animals on current computers [4]. As a result of this limitation some methods were proposed to approximate, or to compute implicitly, the inverse of ${\mathbf {G}}$ [4–6].

Equivalent models that directly estimate SNP effects and that do not rely on ${\mathbf {G}}$, hereafter called ssSNPBLUP, were also proposed [7–9]. However, these models have not yet been implemented and tested on a large scale due to several reasons, such as the lack of breeding value estimation software that is flexible enough to perform ssSNPBLUP, more complicated modeling compared to ssGBLUP, and convergence issues [1]. In Vandenplas et al. [10], we proposed a preconditioned conjugate gradient (PCG) method with a second-level preconditioner that is easy to implement, and that substantially improves the convergence issues associated with two ssSNPBLUP systems. The objective of this paper is to present several computational strategies that improve the efficiency of solving two different ssSNPBLUP systems efficiently with a PCG method. These strategies aim at taking advantage of existing shared-memory parallel libraries while limiting the amount of required random access memory (RAM). Some of these computational strategies can also be implemented in breeding value estimation software that rely on ssGBLUP.

## Methods

### Two ssSNPBLUP systems

In this study, we investigate the ssSNPBLUP linear equations system proposed by Mantysaari and Stranden [11] (ssSNPBLUP_MS) and the ssSNPBLUP linear equations system proposed by Liu et al. [9] (ssSNPBLUP_Liu). The two ssSNPBLUP systems are equivalent and both systems of equations can be summarized as:

$$\begin{aligned} {\mathbf {C}}_{i}{\mathbf {x}}_{i}={\mathbf {b}}_{i}, \end{aligned}$$

where *i* refers to the linear system proposed by Mantysaari and Stranden [11] (*i* = MS) or to the linear system proposed by Liu et al. [9] (*i* = Liu), ${\mathbf {C}}_{i}$ is a symmetric (semi-)definite coefficient matrix, ${\mathbf {x}}_{i}$ is the vector of solutions, and ${\mathbf {b}}_{i}$ is the right-hand side of the linear system.

For simplicity, and without loss of generality, the different matrices and vectors are described below for a univariate animal model. For the linear system of Mantysaari and Stranden [11], $${\mathbf {x}}_{MS}= \left[ \begin{array}{c} \hat{\varvec{\upbeta }}\\ \hat{{\mathbf {u}}}_{n}\\ \hat{{\mathbf {a}}}_{g}\\ \hat{{\mathbf {g}}} \end{array} \right]$$ where $\varvec{\upbeta }$ is the vector of fixed effects, the subscripts *g* and *n* refer to $n_{g}$ genotyped and $n_{n}$ non-genotyped animals, respectively, ${\mathbf {u}}_{n}$ is the vector of additive genetic effects for non-genotyped animals, ${\mathbf {a}}_{g}$ is the vector of residual polygenic effects for genotyped animals, and ${\mathbf {g}}$ is the vector of SNP effects. The vector ${\mathbf {b}}_{MS}$ is equal to $${\mathbf {b}}_{MS}= \left[ \begin{array}{c} {\mathbf {X}}^{'}{\mathbf {R}}^{-1}{\mathbf {y}} \\ {\mathbf {W}}^{'}_{n}{\mathbf {R}}^{-1}_{n}{\mathbf {y}}_{n} \\ {\mathbf {W}}^{'}_{g}{\mathbf {R}}^{-1}_{g}{\mathbf {y}}_{g} \\ {\mathbf {Z}}^{'}{\mathbf {W}}^{'}_{g}{\mathbf {R}}^{-1}_{g}{\mathbf {y}}_{g} \end{array} \right]$$ where ${\mathbf {y}}$ is the vector of records, and the matrices ${\mathbf {X}}$, ${\mathbf {W}}_{n}$ and ${\mathbf {W}}_{g}$ are incidence matrices relating records to the corresponding effects. The matrix ${\mathbf {Z}}$ contains the SNP genotypes (coded as 0 for one homozygous genotype, 1 for the heterozygous genotype, or 2 for the alternate homozygous genotype) centered by their observed means. The matrix $${\mathbf {R}}^{-1}=\left[ \begin{array}{cc} {\mathbf {R}}^{-1}_{n} &{} {\mathbf {0}}\\ {\mathbf {0}} &{} {\mathbf {R}}^{-1}_{g} \end{array} \right]$$ is the inverse of the residual (co)variance structure matrix. The coefficient matrix ${\mathbf {C}}_{MS}$ is equal to:

$$\begin{aligned} {\mathbf {C}}_{MS}= \left[ \begin{array}{cccc} {\mathbf {X}}^{'}{\mathbf {R}}^{-1}{\mathbf {X}} &{} {\mathbf {X}}^{'}_{n}{\mathbf {R}}^{-1}_{n}{\mathbf {W}}_{n} &{} {\mathbf {X}}^{'}_{g}{\mathbf {R}}^{-1}_{g}{\mathbf {W}}_{g} &{} {\mathbf {X}}^{'}_{g}{\mathbf {R}}^{-1}_{g}{\mathbf {W}}_{g}{\mathbf {Z}}\\ {\mathbf {W}}^{'}_{n}{\mathbf {R}}^{-1}_{n}{\mathbf {X}}_{n} &{} {\mathbf {W}}^{'}_{n}{\mathbf {R}}^{-1}_{n}{\mathbf {W}}_{n}+\varvec{\Sigma }_{MS}^{11} &{} \varvec{\Sigma }_{MS}^{12}&{} \varvec{\Sigma }_{MS}^{13}\\ {\mathbf {W}}^{'}_{g}{\mathbf {R}}^{-1}_{g}{\mathbf {X}}_{g} &{} \varvec{\Sigma }_{MS}^{21} &{} {\mathbf {W}}^{'}_{g}{\mathbf {R}}^{-1}_{g}{\mathbf {W}}_{g}+\varvec{\Sigma }_{MS}^{22} &{} {\mathbf {W}}^{'}_{g}{\mathbf {R}}^{-1}_{g}{\mathbf {W}}_{g}{\mathbf {Z}}+\varvec{\Sigma }_{MS}^{23} \\ {\mathbf {Z}}^{'}{\mathbf {W}}^{'}_{g}{\mathbf {R}}^{-1}_{g}{\mathbf {X}}_{g} &{} \varvec{\Sigma }_{MS}^{31} &{} {\mathbf {Z}}^{'}{\mathbf {W}}^{'}_{g}{\mathbf {R}}^{-1}_{g}{\mathbf {W}}_{g} +\varvec{\Sigma }_{MS}^{32} &{} {\mathbf {Z}}^{'}{\mathbf {W}}^{'}_{g}{\mathbf {R}}^{-1}_{g}{\mathbf {W}}_{g}{\mathbf {Z}} +\varvec{\Sigma }_{MS}^{33} \end{array} \right] \end{aligned}$$

where $$\varvec{\Sigma }_{MS}^{-1}= \left[ \begin{array}{ccc} \varvec{\Sigma }_{MS}^{11} &{} \varvec{\Sigma }_{MS}^{12} &{} \varvec{\Sigma }_{MS}^{13} \\ \varvec{\Sigma }_{MS}^{21} &{} \varvec{\Sigma }_{MS}^{22} &{} \varvec{\Sigma }_{MS}^{23} \\ \varvec{\Sigma }_{MS}^{31} &{} \varvec{\Sigma }_{MS}^{32} &{} \varvec{\Sigma }_{MS}^{33} \end{array} \right] = \left[ \begin{array}{ccc} {\mathbf {A}}^{nn} &{} {\mathbf {A}}^{ng} &{} {\mathbf {A}}^{ng}{\mathbf {Z}} \\ {\mathbf {A}}^{gn} &{} \frac{1}{w}{\mathbf {A}}^{gg}+\left( 1-\frac{1}{w} \right) {\mathbf {Q}} &{} {\mathbf {Q}}{\mathbf {Z}} \\ {\mathbf {Z}}^{'}{\mathbf {A}}^{gn} &{} {\mathbf {Z}}^{'}{\mathbf {Q}} &{} {\mathbf {Z}}^{'}{\mathbf {Q}}{\mathbf {Z}}+\frac{m}{1-w}{\mathbf {I}} \end{array} \right] \sigma _{u}^{-2}$$ and where $\sigma _{u}^{-2}$ is the inverse of the additive genetic variance, *w* is the proportion (strictly between 0 and 1) of variance (due to additive genetic effects) considered as residual polygenic effects, and $m=2\sum p_{j}\left( 1-p_{j}\right)$ with $p_{j}$ being the observed allele frequency of the *j*-th SNP. The matrix ${\mathbf {Q}}$ is equal to ${\mathbf {Q}}={\mathbf {A}}^{gn} \left( {\mathbf {A}}^{nn} \right) ^{-1}{\mathbf {A}}^{ng}$, where $${\mathbf {A}}^{-1} = \left[ \begin{array}{cc} {\mathbf {A}}^{nn} &{} {\mathbf {A}}^{ng} \\ {\mathbf {A}}^{gn} &{} {\mathbf {A}}^{gg} \end{array} \right]$$ is the inverse of the pedigree relationship matrix.

For the linear system of Liu et al. [9], $${\mathbf {x}}_{Liu}= \left[ \begin{array}{c} \hat{\varvec{\upbeta }}\\ \hat{{\mathbf {u}}}_{n}\\ \hat{{\mathbf {u}}}_{g}\\ \hat{{\mathbf {g}}} \end{array} \right]$$ where ${\mathbf {u}}_{g}={\mathbf {a}}_{g}+{\mathbf {Z}}{\mathbf {g}}$ is the vector of additive genetic effects for genotyped animals. The vector ${\mathbf {b}}_{Liu}$ is equal to $${\mathbf {b}}_{Liu}= \left[ \begin{array}{c} {\mathbf {X}}^{'}{\mathbf {R}}^{-1}{\mathbf {y}} \\ {\mathbf {W}}^{'}_{n}{\mathbf {R}}^{-1}_{n}{\mathbf {y}}_{n} \\ {\mathbf {W}}^{'}_{g}{\mathbf {R}}^{-1}_{g}{\mathbf {y}}_{g} \\ {\mathbf {0}} \end{array} \right]$$. The coefficient matrix ${\mathbf {C}}_{Liu}$ is equal to:

$$\begin{aligned} {\mathbf {C}}_{Liu}= \left[ \begin{array}{cccc} {\mathbf {X}}^{'}{\mathbf {R}}^{-1}{\mathbf {X}} &{} {\mathbf {X}}^{'}_{n}{\mathbf {R}}^{-1}_{n}{\mathbf {W}}_{n} &{} {\mathbf {X}}^{'}_{g}{\mathbf {R}}^{-1}_{g}{\mathbf {W}}_{g} &{} {\mathbf {0}}\\ {\mathbf {W}}^{'}_{n}{\mathbf {R}}^{-1}_{n}{\mathbf {X}}_{n} &{} {\mathbf {W}}^{'}_{n}{\mathbf {R}}^{-1}_{n}{\mathbf {W}}_{n}+\varvec{\Sigma }_{Liu}^{11} &{} \varvec{\Sigma }_{Liu}^{12}&{} \varvec{\Sigma }_{Liu}^{13}\\ {\mathbf {W}}^{'}_{g}{\mathbf {R}}^{-1}_{g}{\mathbf {X}}_{g} &{} \varvec{\Sigma }_{Liu}^{21} &{} {\mathbf {W}}^{'}_{g}{\mathbf {R}}^{-1}_{g}{\mathbf {W}}_{g}+\varvec{\Sigma }_{Liu}^{22} &{} \varvec{\Sigma }_{Liu}^{23} \\ {\mathbf {0}} &{} \varvec{\Sigma }_{Liu}^{31} &{} \varvec{\Sigma }_{Liu}^{32} &{} \varvec{\Sigma }_{Liu}^{33} \end{array} \right] \end{aligned}$$

where $$\varvec{\Sigma }_{Liu}^{-1}= \left[ \begin{array}{ccc} \varvec{\Sigma }_{Liu}^{11} &{} \varvec{\Sigma }_{Liu}^{12} &{} \varvec{\Sigma }_{Liu}^{13} \\ \varvec{\Sigma }_{Liu}^{21} &{} \varvec{\Sigma }_{Liu}^{22} &{} \varvec{\Sigma }_{Liu}^{23} \\ \varvec{\Sigma }_{Liu}^{31} &{} \varvec{\Sigma }_{Liu}^{32} &{} \varvec{\Sigma }_{Liu}^{33} \end{array} \right] = \left[ \begin{array}{ccc} {\mathbf {A}}^{nn} &{} {\mathbf {A}}^{ng} &{} {\mathbf {0}} \\ {\mathbf {A}}^{gn} &{} {\mathbf {A}}^{gg}+\left( \frac{1}{w}-1 \right) {\mathbf {A}}^{-1}_{gg} &{} -\frac{1}{w}{\mathbf {A}}_{gg}^{-1}{\mathbf {Z}} \\ {\mathbf {0}} &{} -\frac{1}{w}{\mathbf {Z}}^{'}{\mathbf {A}}_{gg}^{-1} &{} \frac{1}{w}{\mathbf {Z}}^{'}{\mathbf {A}}_{gg}^{-1}{\mathbf {Z}}+\frac{m}{1-w}{\mathbf {I}} \end{array} \right] \sigma _{u}^{-2}$$. It is worth noting that ${\mathbf {A}}_{gg}^{-1}={\mathbf {A}}^{gg}-{\mathbf {Q}}$ [12].

### A PCG method

A PCG method is an iterative method that uses successive approximations to obtain more accurate solutions for a linear system at each iteration step [13]. Our implementation of the preconditioned system of linear equations of both ssSNPBLUP has the form:

1 $$\begin{aligned} {\mathbf {D}}^{-1}{\mathbf {M}}^{-1}{\mathbf {C}}{\mathbf {x}}={\mathbf {D}}^{-1}{\mathbf {M}}^{-1}{\mathbf {b}}, \end{aligned}$$

where ${\mathbf {M}}$ is a preconditioner defined below, and ${\mathbf {D}}$ is a second-level diagonal preconditioner proposed by Vandenplas et al. [10] and described in the “Analyses” section.

The main computational costs of the PCG method for solving ssSNPBLUP systems are the computation of some terms of the preconditioner ${\mathbf {M}}$ and the multiplication of the coefficient matrix ${\mathbf {C}}$ by a vector at each PCG iteration. In the next section, we propose computational approaches at approximating specific elements of ${\mathbf {M}}$ and to multiply ${\mathbf {C}}$ by a vector in an efficient manner.

### Computation of the preconditioner ${\mathbf {M}}$

In animal breeding, a (block-)diagonal preconditioner is commonly used [14]. The (block-)diagonal elements of matrices, such as ${\mathbf {X}}^{'}{\mathbf {R}}^{-1}{\mathbf {X}}$ and ${\mathbf {W}}^{'}_{n}{\mathbf {R}}^{-1}_{n}{\mathbf {W}}_{n}+\varvec{\Sigma }^{11}_{i}$ (*i* = MS, Liu), can be easily obtained, in contrast to ${\mathbf {W}}^{'}_{g}{\mathbf {R}}^{-1}_{g}{\mathbf {W}}_{g}+\varvec{\Sigma }^{22}_{i}$ and ${\mathbf {Z}}^{'}{\mathbf {W}}^{'}_{g}{\mathbf {R}}^{-1}_{g}{\mathbf {W}}_{g}{\mathbf {Z}} +\varvec{\Sigma }^{33}_{MS}$ which contain terms like ${\mathbf {A}}_{gg}^{-1}$, ${\mathbf {Z}}{'}{\mathbf {A}}_{gg}^{-1}{\mathbf {Z}}$, or ${\mathbf {Z}}{'}{\mathbf {Q}}{\mathbf {Z}}$. Since the preconditioner aims to approximate the coefficient matrix, we approximate $diag\left( {\mathbf {A}}_{gg}^{-1}\right)$ with a Monte Carlo approach based on 1000 samples, as proposed by Masuda et al. [15]. Furthermore, the *j*-th diagonal element of $diag\left( {\mathbf {Z}}^{'}{\mathbf {A}}_{gg}^{-1}{\mathbf {Z}}\right)$ was approximated to $2 n_{g} p_{j} (1-p_{j})$, and the *j*-th diagonal element of $diag\left( {\mathbf {Z}}^{'}{\mathbf {Q}}{\mathbf {Z}}\right)$ was approximated to $\left( 2 n_{g} + n_{offspring} \right) p_{j} \left( 1 - p_{j} \right)$, where $n_{offspring}$ is the total number of offspring of all the $n_{g}$ genotyped animals (see Additional file 1 for derivations). These approximations always provided the same convergence rate compared with the exact values (results not shown).

### Computational strategies for the multiplication of ${\mathbf {C}}$ by a vector

Our approach for the efficient multiplication of ${\mathbf {C}}$ by a vector, e.g. ${\mathbf {x}}$, relies on splitting the coefficient matrix ${\mathbf {C}}$ into multiple parts for which the multiplication by a vector is easier to perform.

For ssSNPBLUP_MS, the coefficient matrix ${\mathbf {C}}_{MS}$ can be split into:

2 $$\begin{aligned} {\mathbf {C}}_{MS} = {\mathbf {T}}^{'}\left( {\mathbf {C}}_{MS_{LS}}+{\mathbf {C}}_{MS_{R1}}\right) {\mathbf {T}}+{\mathbf {C}}_{MS_{R2}} \end{aligned}$$

with $${\mathbf {T}}= \left[ \begin{array}{cccc} {\mathbf {I}} &{}{\mathbf {0}} &{}{\mathbf {0}} &{}{\mathbf {0}} \\ {\mathbf {0}} &{}{\mathbf {I}} &{}{\mathbf {0}} &{}{\mathbf {0}} \\ {\mathbf {0}} &{}{\mathbf {0}} &{}{\mathbf {I}} &{}{\mathbf {0}} \\ {\mathbf {0}} &{}{\mathbf {0}} &{}{\mathbf {0}} &{}{\mathbf {Z}} \end{array} \right]$$, $${\mathbf {C}}_{MS_{LS}}= \left[ \begin{array}{cccc} {\mathbf {X}}^{'}{\mathbf {R}}^{-1}{\mathbf {X}} &{} {\mathbf {X}}^{'}_{n}{\mathbf {R}}^{-1}_{n}{\mathbf {W}}_{n} &{} {\mathbf {X}}^{'}_{g}{\mathbf {R}}^{-1}_{g}{\mathbf {W}}_{g} &{} {\mathbf {X}}^{'}_{g}{\mathbf {R}}^{-1}_{g}{\mathbf {W}}_{g}\\ {\mathbf {W}}^{'}_{n}{\mathbf {R}}^{-1}_{n}{\mathbf {X}}_{n} &{} {\mathbf {W}}^{'}_{n}{\mathbf {R}}^{-1}_{n}{\mathbf {W}}_{n}&{} {\mathbf {0}}&{} {\mathbf {0}}\\ {\mathbf {W}}^{'}_{g}{\mathbf {R}}^{-1}_{g}{\mathbf {X}}_{g} &{} {\mathbf {0}}&{} {\mathbf {W}}^{'}_{g}{\mathbf {R}}^{-1}_{g}{\mathbf {W}}_{g}&{} {\mathbf {W}}^{'}_{g}{\mathbf {R}}^{-1}_{g}{\mathbf {W}}_{g} \\ {\mathbf {W}}^{'}_{g}{\mathbf {R}}^{-1}_{g}{\mathbf {X}}_{g} &{} {\mathbf {0}}&{} {\mathbf {W}}^{'}_{g}{\mathbf {R}}^{-1}_{g}{\mathbf {W}}_{g} &{} {\mathbf {W}}^{'}_{g}{\mathbf {R}}^{-1}_{g}{\mathbf {W}}_{g} \end{array} \right]$$, $${\mathbf {C}}_{MS_{R1}}= \left[ \begin{array}{cccc} {\mathbf {0}} &{}{\mathbf {0}} &{}{\mathbf {0}} &{}{\mathbf {0}} \\ {\mathbf {0}} &{}{\mathbf {0}}&{} {\mathbf {0}} &{} {\mathbf {A}}^{ng} \\ {\mathbf {0}} &{}{\mathbf {0}} &{} \left( 1-\frac{1}{w} \right) {\mathbf {Q}} &{} {\mathbf {Q}} \\ {\mathbf {0}} &{}{\mathbf {A}}^{gn} &{} {\mathbf {Q}} &{} {\mathbf {Q}} \end{array} \right] \sigma _{u}^{-2}$$, and $${\mathbf {C}}_{MS_{R2}}= \left[ \begin{array}{cccc} {\mathbf {0}} &{}{\mathbf {0}} &{}{\mathbf {0}} &{}{\mathbf {0}} \\ {\mathbf {0}} &{}{\mathbf {A}}^{nn} &{} {\mathbf {A}}^{ng} &{} {\mathbf {0}} \\ {\mathbf {0}} &{}{\mathbf {A}}^{gn} &{} \frac{1}{w}{\mathbf {A}}^{gg}&{} {\mathbf {0}} \\ {\mathbf {0}} &{}{\mathbf {0}} &{} {\mathbf {0}}&{} \frac{m}{1-w}{\mathbf {I}} \end{array} \right] \sigma _{u}^{-2}$$.

The multiplication of ${\mathbf {C}}_{MS}$ by a vector, e.g. ${\mathbf {x}}_{MS}$, can be easily computed in multiple steps as follows:

$$\begin{aligned} {\mathbf {C}}_{MS}{\mathbf {x}}_{MS} = {\mathbf {T}}^{'}\left[ \left[ {\mathbf {C}}_{LS}{\mathbf {v}}_{1} \right] + \left[ {\mathbf {C}}_{MS_{R1}}{\mathbf {v}}_{1} \right] \right] + \left[ {\mathbf {C}}_{MS_{R2}}{\mathbf {x}}_{MS} \right] \end{aligned}$$

where the brackets $\left[ . \right]$ indicate the order of the matrix-vector operations, and $${\mathbf {v}}_{1} = {\mathbf {T}}{\mathbf {x}}_{MS} = \left[ \begin{array}{c} \hat{{\mathbf {b}}}\\ \hat{{\mathbf {u}}}_{n}\\ \hat{{\mathbf {a}}}_{g}\\ {\mathbf {Z}}\hat{{\mathbf {g}}} \end{array} \right]$$.

For ssSNPBLUP_Liu, the multiplication of the coefficient matrix ${\mathbf {C}}_{Liu}$ by a vector, e.g. ${\mathbf {x}}_{Liu}$, can be performed in multiple steps as:

3 $$\begin{aligned} {\mathbf {C}}_{Liu}{\mathbf {x}}_{Liu}={\mathbf {C}}_{Liu_{LS}}{\mathbf {x}}_{Liu}+{\mathbf {T}}^{'}{\mathbf {C}}_{Liu_{R1}}{\mathbf {T}}{\mathbf {x}}_{Liu}+{\mathbf {C}}_{Liu_{R2}}{\mathbf {x}}_{Liu} \end{aligned}$$

with $${\mathbf {C}}_{Liu_{LS}}= \left[ \begin{array}{cccc} {\mathbf {X}}^{'}{\mathbf {R}}^{-1}{\mathbf {X}} &{} {\mathbf {X}}^{'}_{n}{\mathbf {R}}^{-1}_{n}{\mathbf {W}}_{n} &{} {\mathbf {X}}^{'}_{g}{\mathbf {R}}^{-1}_{g}{\mathbf {W}}_{g} &{} {\mathbf {0}}\\ {\mathbf {W}}^{'}_{n}{\mathbf {R}}^{-1}_{n}{\mathbf {X}}_{n} &{} {\mathbf {W}}^{'}_{n}{\mathbf {R}}^{-1}_{n}{\mathbf {W}}_{n}&{} {\mathbf {0}}&{} {\mathbf {0}}\\ {\mathbf {W}}^{'}_{g}{\mathbf {R}}^{-1}_{g}{\mathbf {X}}_{g} &{} {\mathbf {0}} &{} {\mathbf {W}}^{'}_{g}{\mathbf {R}}^{-1}_{g}{\mathbf {W}}_{g}&{} {\mathbf {0}} \\ {\mathbf {0}} &{} {\mathbf {0}} &{} {\mathbf {0}} &{} {\mathbf {0}} \end{array} \right]$$, $${\mathbf {C}}_{Liu_{R1}} = \left[ \begin{array}{cccc} {\mathbf {0}} &{} {\mathbf {0}}&{} {\mathbf {0}}&{} {\mathbf {0}} \\ {\mathbf {0}} &{} {\mathbf {0}} &{} {\mathbf {0}} &{} {\mathbf {0}} \\ {\mathbf {0}} &{} {\mathbf {0}} &{} \left( \frac{1}{w}-1 \right) {\mathbf {A}}^{-1}_{gg} &{} -\frac{1}{w}{\mathbf {A}}_{gg}^{-1} \\ {\mathbf {0}} &{} {\mathbf {0}} &{} -\frac{1}{w}{\mathbf {A}}_{gg}^{-1} &{} \frac{1}{w}{\mathbf {A}}_{gg}^{-1} \end{array} \right] \sigma _{u}^{-2}$$, and $${\mathbf {C}}_{Liu_{R2}} = \left[ \begin{array}{cccc} {\mathbf {0}} &{} {\mathbf {0}}&{} {\mathbf {0}}&{} {\mathbf {0}} \\ {\mathbf {0}} &{} {\mathbf {A}}^{nn} &{} {\mathbf {A}}^{ng} &{} {\mathbf {0}} \\ {\mathbf {0}} &{} {\mathbf {A}}^{gn} &{} {\mathbf {A}}^{gg} &{} {\mathbf {0}} \\ {\mathbf {0}} &{} {\mathbf {0}} &{} {\mathbf {0}} &{} \frac{m}{1-w}{\mathbf {I}} \end{array} \right] \sigma _{u}^{-2}$$.

It is worth noting that the multiplication of ${\mathbf {C}}_{MS_{LS}}$ and of ${\mathbf {C}}_{Liu_{LS}}$ by a vector can be performed with approaches that have already been developed in animal breeding, such as iteration-on-data approaches [16–19], because these matrices are similar to those obtained with traditional pedigree BLUP. Similarly, the multiplication of ${\mathbf {C}}_{MS_{R2}}$ and of ${\mathbf {C}}_{Liu_{R2}}$ (both involving ${\mathbf {A}}^{-1}$) by a vector, can be easily computed using strategies such as those developed by Stranden and Lidauer [18], or as described below.

In the following, we describe in detail computational strategies for multiplying efficiently submatrices of ${\mathbf {T}}$, of its transpose, of ${\mathbf {C}}_{MS_{R1}}$, of ${\mathbf {C}}_{MS_{R2}}$, of ${\mathbf {C}}_{Liu_{R1}}$, and of ${\mathbf {C}}_{Liu_{R2}}$, by a vector. It should be noted that the multiplication of these matrices requires the multiplication of the centered genotype matrix ${\mathbf {Z}}$, its transpose $\mathbf {Z'}$, ${\mathbf {Q}}$, and ${\mathbf {A}}_{gg}^{-1}$, by an array. Furthermore, while the proposed computational strategies are described in the context of a univariate animal model, they are readily applicable to more complex models, such as multivariate maternal models (see Additional file 2 for a description of a ssSNPBLUP_MS system associated with a standard bivariate maternal model).

#### Multiplication of ${\mathbf {C}}_{i_{LS}}$ by a vector

The implemented approach for multiplying ${\mathbf {C}}_{i_{LS}}$ (*i* = MS, Liu) by a vector was the three-step approach combined with an iteration-on-data technique, as proposed by Stranden and Lidauer [18]. The phenotypes and associated levels for all effects were stored in RAM to allow shared-memory parallelization. Phenotypes were stored using double precision reals, and levels for all effects were stored using 4-byte integers. Each thread was associated with a same amount of records to make the computations involving submatrices of ${\mathbf {C}}_{i_{LS}}$ as even as possible across the threads. Furthermore, the records were sorted following an increasing order of the effect with the largest number of levels to minimize RAM required by the temporary arrays.

#### Multiplication of ${\mathbf {Z}}$, or $\mathbf {Z'}$, by an array

The main cost of the multiplication of the matrix ${\mathbf {T}}$, or its transpose, by an array is the multiplication of the centered genotyped matrix ${\mathbf {Z}}$, or its transpose $\mathbf {Z'}$, by an array.

To benefit from shared-memory parallel programming while limiting the amount of RAM required, the SNP genotypes included in ${\mathbf {Z}}$ were stored in RAM using the Plink 1 binary form [20]. In brief, the value of each SNP locus (coded as 0 for one homozygous genotype, 1 for the heterozygous genotype, 2 for the alternate homozygous genotype, or missing) is coded using 2 bits, and each byte (B) stores the genotype of four genotyped animals for a same SNP (see [20] for more details). Observed allele frequencies needed for centering SNP genotypes were stored into a double precision real array. This approach requires $\frac{n_{g}*n_{SNP}}{4}$ B to store the genotypes and $8n_{SNP}$ B to store the allele frequencies. For example, to store one million genotypes with 50,000 SNPs, this approach requires around 12 GB RAM. In comparison, the storage of the same information using a double precision real array would require 32 times more RAM, i.e. around 373 GB.

Because the matrix ${\mathbf {Z}}$ is stored in Plink 1 binary form in RAM, a custom implementation of a Matrix-Matrix product is needed. The matrix ${\mathbf {Z}}$ is split into small blocks intended to fit into the CPU cache. Each block of ${\mathbf {Z}}$ is converted into a small matrix of double-precision numbers (corresponding to centered genotypes or zero for missing values) and subsequently multiplied with part of the array. This implementation uses vectorization and loop unrolling to make optimal use of available hardware resources on modern CPUs.

While it might not be straightforward to implement, the proposed approach for multiplying ${\mathbf {Z}}$, or its transpose, by an array could be also used in single-step evaluations that rely on genomic relationship matrices. Indeed, the multiplication of the inverse of the genomic relationship matrix by an array could be replaced by a system of equations that would be solved iteratively and that would require the multiplication of ${\mathbf {Z}}$, and its transpose, by an array [21].

#### Multiplication of ${\mathbf {A}}^{-1}$ by an array

The multiplication of the matrices ${\mathbf {C}}_{MS_{R2}}$, and ${\mathbf {C}}_{Liu_{R2}}$, by an array requires the multiplication of ${\mathbf {A}}^{-1}$ by an array. Due to the small amounts of RAM available in the past, an approach that only requires reading the pedigree was developed to multiply ${\mathbf {A}}^{-1}$ by an array [16]. While such an approach is memory-efficient, it does not allow an efficient shared-memory parallelization of the multiplication of ${\mathbf {A}}^{-1}$ by an array.

With the current large amounts of RAM available, it is now possible to store ${\mathbf {A}}^{-1}$ in RAM, even for large pedigrees. For our implementation, since ${\mathbf {A}}^{-1}$ is a sparse and symmetric matrix, its upper triangular part is stored in RAM using the well-known and widely used 3-array variation of the compressed row storage (CRS3) format [13]. The CRS3 format of a sparse matrix is specified by two arrays of (4-byte) integers (named *IA* and *JA*) and one (double precision) real array (named *AA*). The array *IA*, of size equal to the number of rows of the sparse matrix plus one, contains the pointers to the beginning of each row of the sparse matrix in the arrays *JA* and *AA*. The array *JA*, of size equal to the number of non-zero real values, contains the column indices of the corresponding elements stored in *AA*. The array *AA* contains the non-zero real values of the sparse matrix [13].

Following Henderson’s rules to construct ${\mathbf {A}}^{-1}$ recursively [22], adding the contributions of one animal to ${\mathbf {A}}^{-1}$ leads to adding three diagonal elements and three off-diagonal elements to the upper-triangular part of ${\mathbf {A}}^{-1}$. Therefore, assuming that there are *n* animals in the pedigree, the maximum number of non-zeros elements in the upper-triangular part of ${\mathbf {A}}^{-1}$ is equal to 4*n* (that is, the sum of *n* diagonal elements and of 3*n* off-diagonal elements). With the CRS3 format, an upper bound of RAM needed to store the upper triangular part of ${\mathbf {A}}^{-1}$ as a sparse matrix using double precision reals is equal to the sum of $4*(n+1)$ B for the array *IA*, of $4*4n$ B for the array *JA*, and of $8*4n$ B for the array *AA*, which is equal to a total of $52n+4$ B. This upper bound increases linearly as the number of animals increases in the pedigree, and is equal, for example, to 1.45 GB for a pedigree with 30 million animals. Using shared-memory parallel programming, efficient libraries, such as sparse BLAS routines, can be used for multiplying ${\mathbf {A}}^{-1}$ by an array.

#### Multiplication of ${\mathbf {Q}}$ by an array

The multiplication of ${\mathbf {C}}_{MS_{R1}}$ by an array implies several multiplications of the matrix ${\mathbf {Q}}$ by an array, and subsequently several multiplications of $\left( {\mathbf {A}}^{nn}\right) ^{-1}$ by an array. This matrix $\left( {\mathbf {A}}^{nn}\right) ^{-1}$ has a size almost equal to the number of animals in the pedigree, because, for most single-step genomic evaluations, the number of genotyped animals is a small fraction of the number of animals in the pedigree. An alternative computation of the matrix ${\mathbf {Q}}$ is as follows (see Additional file 3 for the derivation):

$$\begin{aligned} {\mathbf {Q}} = {\mathbf {A}}^{gn}_{anc}\left( {\mathbf {A}}^{gg}_{anc}\right) ^{-1}{\mathbf {A}}^{ng}_{anc}+\varvec{\Delta } \end{aligned}$$

with the matrices ${\mathbf {A}}^{ii}_{anc}$ being submatrices of the inverse of the pedigree relationship matrix that include only the genotyped animals and their ancestors, and the matrix $\varvec{\Delta }$ being equal to $\varvec{\Delta }={\mathbf {A}}^{gg} - {\mathbf {A}}^{gg}_{anc}$.

Based on Henderson’s rules [22] to directly construct ${\mathbf {A}}^{-1}$, it follows that the matrix $\varvec{\Delta }$ contains only the contributions of the non-genotyped offspring of the genotyped animals that are not ancestors of genotyped animals (see Additional file 3 for details). Therefore, the matrix $\varvec{\Delta }$ can be easily and directly constructed by reading the pedigree only once.

Finally, it is worth noting that the multiplication of ${\mathbf {C}}_{MS_{R1}}$ by a vector involves four multiplications of ${\mathbf {Q}}$ by an array. However, only two multiplications of ${\mathbf {Q}}$ by an array are actually required due to the presence of the same multiplications.

#### Multiplication of ${\mathbf {A}}_{gg}^{-1}$ by an array

The multiplication of ${\mathbf {C}}_{Liu_{R1}}$ by a vector implies two multiplications of the matrix ${\mathbf {A}}_{gg}^{-1}$ by an array. As proposed by Stranden et al. [12], the multiplication of ${\mathbf {A}}_{gg}^{-1}$ by an array is performed using sparse matrices:

$$\begin{aligned} {\mathbf {A}}_{gg}^{-1} = {\mathbf {A}}^{gg}_{anc}-{\mathbf {A}}^{gn}_{anc}\left( {\mathbf {A}}^{nn}_{anc}\right) ^{-1}{\mathbf {A}}^{ng}_{anc} . \end{aligned}$$

The sparse matrices ${\mathbf {A}}^{gg}_{anc}$, ${\mathbf {A}}^{gn}_{anc}$, and ${\mathbf {A}}^{nn}_{anc}$, were stored in RAM to enable shared-memory parallelization.

### Data

The implementations of ssSNPBLUP as described in the previous sections were compared to each other in terms of computational efficiency. This comparison was based on data and associated variance components from the bivariate routine genetic evaluation published in April 2019 for livability of calves for the Netherlands and the Flemish region in Belgium [23, 24]. The data file included 25,184,654 calf records. The pedigree included 29,885,286 animals. The genotypes included 37,995 segregating SNPs, and were associated with 131,189 animals without phenotypes and with 129,402 animals with phenotypes.

The two traits are livability of calves born from heifers, and livability of calves born from multiparous cows. The bivariate mixed model included random effects (correlated additive direct and maternal genetic effects, permanent environmental effect and residual), fixed co-variables ((direct and maternal) heterosis and recombination effects) and fixed cross-classified effects (herd x year x season, year x month, age at calving, and parity). More details about the model and genetic parameters can be found in [23] and [24].

For both ssSNPBLUP_Liu and ssSNPBLUP_MS, the observed allele frequencies were used to center the genotype matrix, and the compatibility between pedigree and genomic information was guaranteed by fitting two ${\mathbf {J}}$ covariates (corresponding to the additive and maternal genetic effects) as fixed effects in the model [25]. The proportion of variance (due to additive genetic effects) considered as residual polygenic effects, *w*, was assumed to be equal to 0.05.

### Analyses

Both ssSNPBLUP_MS and ssSNPBLUP_Liu were solved by using a Fortran 2003 program that implements the described computational approaches. The program also exploits BLAS and sparse BLAS routines, the parallel direct sparse solver PARDISO, all from the multi-threaded Intel Math Kernel Library 11.3.2, and OpenMP parallel computing. Except for the preconditioner, all real vectors and matrices were stored using double precision reals. For comparison, ssSNPBLUP_Liu was also performed with the centered genotyped matrix stored in RAM using double precision reals, instead of the Plink 1 binary form.

In this study, the preconditioner is defined for both ssSNPBLUP as:

$$\begin{aligned} {\mathbf {M}}= \left[ \begin{array}{ccc} diag\left( {\mathbf {C}}_{f1,f1}\right) &{} {\mathbf {0}}&{} {\mathbf {0}}\\ {\mathbf {0}} &{} block\_diag \left( {\mathbf {C}}_{rr} \right) &{} {\mathbf {0}} \\ {\mathbf {0}} &{} {\mathbf {0}} &{} {\mathbf {C}}_{f2,f2} + 10^{-4}* diag \left( {\mathbf {C}}_{f2,f2} \right) \end{array} \right] \end{aligned}$$

where the subscripts *f1*, *f2*, and *r* refer to the equations associated with the herd x year x season effect, the other fixed effects, and the random effects, respectively, and $block\_diag \left( {\mathbf {C}}_{rr} \right)$ is a block-diagonal matrix with blocks corresponding to equations for different traits within a level (e.g. an animal). The diagonal and block-diagonal elements of the preconditioner were stored using single precision reals, while the matrix ${\mathbf {C}}_{f2,f2} + 10^{-4}* diag \left( {\mathbf {C}}_{f2,f2} \right)$ was stored using the CRS3 format described earlier.

The diagonal elements of the second-level diagonal preconditioner ${\mathbf {D}}$ that correspond to the equations of the direct and maternal effects of the SNP effects were equal to $10^{3}$ for ssSNPBLUP_MS, and $10^{2}$ for ssSNPBLUP_Liu [10]. Other diagonal elements were equal to 1.

For both ssSNPBLUP systems, convergence was achieved when $\frac{\Vert {\mathbf {r}}_{i,k} \Vert }{\Vert {\mathbf {b}}_{i} \Vert } < 10^{-6}$ with $\Vert . \Vert$ being the 2-norm, and ${\mathbf {r}}_{i,k}$ being the residual after *k+1* iterations computed as ${\mathbf {r}}_{i,k}={\mathbf {b}}_{i}-{\mathbf {C}}_{i}{\mathbf {x}}_{i,k}$, although it is not strictly comparable across systems. For all systems, the smallest and largest eigenvalues of the preconditioned coefficient matrices ${\mathbf {D}}^{-1}{\mathbf {M}}^{-1}{\mathbf {C}}$ that influence the convergence of the PCG method were estimated using the Lanczos method based on information obtained from the PCG method [26]. Effective condition numbers were computed from the ratio of these estimates, as this provides an indication of the properties of the preconditioned system of equations, with higher effective spectral condition numbers being associated with poorer convergence [27].

All computations were performed on a computer with 528 GB and running RedHat 7.4 (x86_64) with an Intel Xeon E5-2667 (3.20 GHz) processor with 16 cores. The number of OpenMP threads used for all computations was equal to 5. All reported times are indicative, because they may have been influenced by other jobs running simultaneously on the computer.

## Results

Characteristics and results for different parts of the preparation and solving steps for ssSNPBLUP_MS and ssSNPBLUP_Liu using the Plink 1 binary form, or using double precision reals, are in Tables 1 and 2. All three ssSNPBLUP systems included 142,283,778 equations. Estimates for all fixed effects, additive direct and maternal genetic effects, and other random effects, of the three ssSNPBLUP systems were (almost) the same after convergence was reached (e.g., the Pearson correlations between all estimates for the direct and maternal genetic effects of the three systems were higher than 0.999).

**Table 1:** Characteristics of different ssSNPBLUP systems

Characteristic	ssSNPBLUP_Liu (Plink)^a^	ssSNPBLUP_Liu (DP)^b^	ssSNPBLUP_MS^c^
Number of iterations	3,358	3,359	6,334
Smallest eigenvalue	$2.304*10^{-6}$	$2.304*10^{-6}$	$1.989*10^{-6}$
Largest eigenvalue	3.813	3.813	5.194
Spectral condition number	$1.655*10^{6}$	$1.655*10^{6}$	$2.612*10^{6}$
Software peak memory (MB)^d^	18,120.7	89,615.7	27,780.3

^a^ssSNPBLUP model proposed by Liu et al. [9] and using the Plink 1 binary form; or ^b^ using double precision reals; ^c^ssSNPBLUP model proposed by Mantysaari and Stranden [11] and using the Plink 1 binary form; ^d^The software peak memory is defined as the peak resident size (VmHWM) obtained from the Linux /proc virtual file system

**Table 2:** Wall clock times for the preparation and solving processes of different ssSNPBLUP systems

Wall clock time (s)	ssSNPBLUP_Liu (Plink)^a^	ssSNPBLUP_Liu (DP)^b^	ssSNPBLUP_MS^c^
$diag \left( {\mathbf {A}}_{gg}^{-1} \right)$^d^	136.69	139.78	136.20
Preconditioner^d^	546.41	581.42	1,177.26
${\mathbf {A}}^{-1}$^d^	50.62	57.40	50.77
${\mathbf {Z}}{\mathbf {v}}$^e^	3.47	7.77	3.42
$\mathbf {Z'}{\mathbf {v}}$^e^	1.53	4.61	1.45
Average time/iteration	12.82	20.23	16.89
Iterative process	43,074.48	67,961.84	107,041.71
Software total time	44,531.00	69,593.09	109,126.07

^a^ssSNPBLUP model proposed by Liu et al. [9] and using the Plink 1 binary form; or ^b^using double precision reals; ^c^ssSNPBLUP model proposed by Mantysaari and Stranden [11] and using the Plink 1 binary form; ^d^Wall clock time needed for the computation of the mentioned matrix; ^e^Multiplication of the centered genotype matrix, or its transpose, by an array

The wall clock time spent outside the iterative process varied between 1456 s for ssSNPBLUP_Liu using the Plink 1 binary form and 2084 s for ssSNPBLUP_MS. Those times include input/output operations and computations of several matrices. For example, the computation of the diagonal elements of the matrix ${\mathbf {A}}_{gg}^{-1}$ using a Monte Carlo method [15] required less than 140 s for each of the three evaluations (Table 2). As described by Masuda et al. [15], the Monte Carlo method only requires ${\mathbf {A}}^{gg}_{anc}$, ${\mathbf {A}}^{gn}_{anc}$, and ${\mathbf {A}}^{nn}_{anc}$. These three sparse matrices were computed using the pedigree of the 558,642 ancestors of the 260,591 genotyped animals. Also, the preparation of ${\mathbf {A}}^{-1}$ for the whole pedigree, i.e. for the 29,885,286 animals, required less than a minute (Table 2) and about 1.40 GB RAM. Finally, while the same amount of RAM (i.e. 807.71 MB) was required across the three evaluations, the computation of the preconditioner ${\mathbf {M}}$ for ssSNPBLUP_MS needed about twice the wall clock time of the computation of ${\mathbf {M}}$ for ssSNPBLUP_Liu (Table 2). This was due to the fact that the diagonal elements of ${\mathbf {Z}}^{'}{\mathbf {W}}^{'}_{g}{\mathbf {R}}^{-1}_{g}{\mathbf {W}}_{g}{\mathbf {Z}}$ were computed explicitly for ssSNPBLUP_MS. This additional computation also explains the additional wall clock time needed for ssSNPBLUP_MS outside the iterative process.

**Fig. 1:**
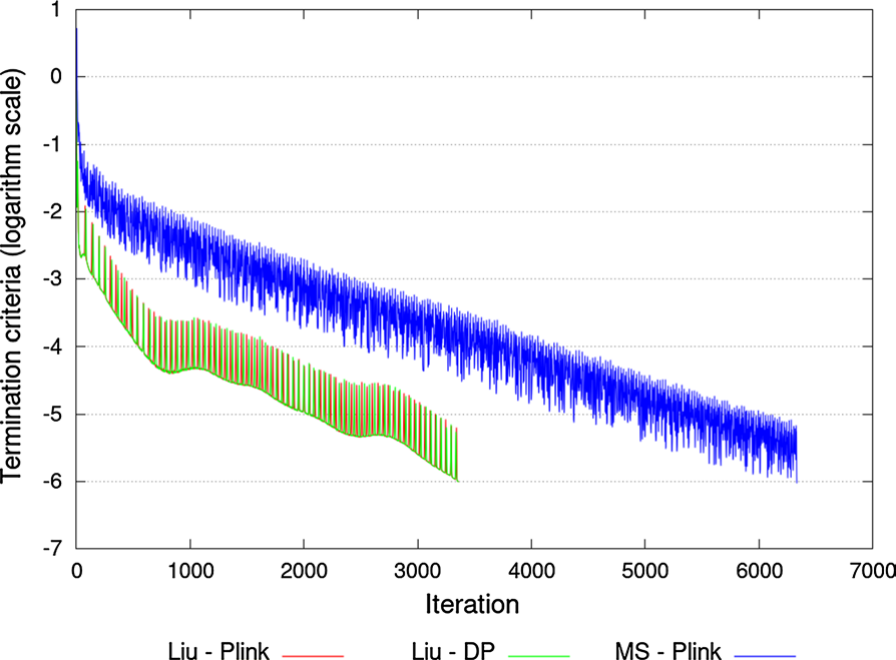
Termination criteria for different ssSNPBLUP systems. The three systems investigated were a ssSNPBLUP system proposed by Liu et al. [9] using the Plink 1 binary form, or using double precision (DP) reals, and a ssSNPBLUP proposed by Mantysaari and Stranden [11] using the Plink 1 binary form

As expected, ssSNPBLUP_Liu using the Plink 1 binary form and ssSNPBLUP_Liu using double precision reals, converged in about the same number of iterations (i.e. around 3360 iterations; Fig.  1; Table 1). Their preconditioned coefficient matrices had an effective spectral condition number equal to $1.655*10^{6}$, resulting from the same extreme eigenvalues (Table 1). Differences between the two ssSNPBLUP_Liu were observed at the level of their performances. ssSNPBLUP_Liu using the Plink 1 binary form required a maximum of around 18 GB RAM and about 13 s per iteration. In comparison, ssSNPBLUP_Liu using double precision reals required a maximum of around 89 GB RAM and about 20 s per iteration (Tables 1 and 2). The increase in RAM was due to the fact that the centered genotyped matrix stored with double precision reals required about 74 GB RAM (versus <3 GB RAM with the Plink 1 binary form). The increase in time per iteration was due to the fact that the wall clock time for the multiplication of the centered genotyped matrix by an array using the Intel MKL DGEMM subroutine was more than twice the wall clock time needed for the same multiplication using our subroutine with the Plink 1 binary form (Table 2). Due to this increase in time per iteration, ssSNPBLUP_Liu using double precision reals needed about 56% more wall clock time to complete than ssSNPBLUP_Liu using Plink 1 binary form (that required about 12 h to complete) (Table 2). Using the Plink 1 binary form instead of double precision reals to store the genotype matrix in-memory is therefore beneficial for both memory and time requirements.

In comparison to ssSNPBLUP_Liu using the Plink 1 binary form, ssSNPBLUP_MS using the Plink 1 binary form was less efficient in terms of convergence, wall clock time, and RAM (Figure 1; Tables 1 and 2). The PCG method required a total of 6334 iterations to reach convergence, which can be partly explained by a larger spectral condition number, equal to $2.612*10^{6}$. Previously Vandenplas et al. [10] noted that spectral condition numbers and convergence of the PCG method for ssSNPBLUP_MS are worse than for ssSNPBLUP_Liu. Furthermore, ssSNPBLUP_MS required 5 additional seconds per iteration in comparison to ssSNPBLUP_Liu using the Plink 1 binary form (Table 2). This additional time per iteration is mainly due to additional computations needed for ssSNPBLUP_MS when multiplying ${\mathbf {C}}_{MS_{LS}}$ and ${\mathbf {C}}_{MS_{R1}}$ by a vector. For ${\mathbf {C}}_{MS_{LS}}$, compared to ${\mathbf {C}}_{Liu_{LS}}$, this was due to the additional non-zero entries for the SNP equations. For ${\mathbf {C}}_{MS_{R1}}$, compared to ${\mathbf {C}}_{Liu_{R1}}$, the extra time needed was mainly due to the presence of ${\mathbf {A}}^{ng}$ and its transpose. The larger number of iterations to reach convergence and the longer time per iteration are the two main reasons that explain that ssSNPBLUP_MS completed in almost three times the time needed for ssSNPBLUP_Liu using the Plink 1 binary form. Finally, ssSNPBLUP_MS also required more RAM than ssSNPBLUP_Liu (around 52% more) due to additional temporary arrays to perform the multiplication of the Eq. (2).

## Discussion

In this study, several computational strategies were proposed to compute a preconditioner ${\mathbf {M}}$ for different ssSNPBLUP systems and to multiply the associated coefficient matrix ${\mathbf {C}}$ by a vector efficiently. The different strategies are based on approximations for the computation of the preconditioner, and on the splitting of the coefficient matrix ${\mathbf {C}}$ into multiple parts. Some matrices, such as ${\mathbf {A}}^{-1}$, are also computed explicitly and stored in RAM to enable the use of efficient parallel libraries (e.g. BLAS and sparse BLAS). We also developed an approach to multiply a centered genotype matrix by an array when the genotype matrix is stored using a Plink 1 binary form. In general, it is not possible to write a matrix-matrix product subroutine that outperforms a good BLAS DGEMM implementation like the one found in the Intel MKL by a significant margin, if at all. We have shown however that significantly better performance can be achieved by storing the genotype matrix in a compressed form and applying the computation directly to that form.

Across the three implemented evaluations, ssSNPBLUP_Liu using the Plink 1 binary form outperformed the two others in terms of RAM and time requirements. Regarding RAM requirements, the main gain can be explained by the use of the Plink 1 binary form. Assuming one million genotypes of 50,000 SNPs, using the Plink 1 binary form would require around 12 GB to store the genotype matrix, while using double precision reals would require around 373 GB. Even with dimensionality-reduction methods [6, 28], single-step evaluations will still require more RAM than with the Plink 1 binary form. For example, assuming that 20,000 eigenvalues explain 99% of the variation of the genomic information, around 149 GB would still be needed to store the reduced genotype matrix. Similar amounts of RAM would also be required for single-step evaluations using dosage scores (e.g. to account for imputation errors [29, 30]), or based on the algorithm for proven and young animals [4] or on the Woodbury decomposition of the genomic relationship matrix [5], because these approaches require real arrays. Therefore, for a same amount of RAM, ssSNPBLUP using the Plink 1 binary form allows more genotyped animals in a single-step evaluation than the other approaches. A second reason of smaller RAM requirements by ssSNPBLUP_Liu is that in our implementation fewer temporary arrays were needed for ssSNPBLUP_Liu than for ssSNPBLUP_MS.

Regarding the time requirements of the different approaches implemented, ssSNPBLUP_Liu using the Plink 1 binary form used the smallest amount of time per iteration due to its use of the Plink 1 binary form and to fewer multiplications needed than ssSNPBLUP_MS. In addition, the convergence properties of ssSNPBLUP_Liu are better than those of ssSNPBLUP_MS [10, 31]. Hence, it is preferable to implement ssSNPBLUP_Liu instead of ssSNPBLUP_MS. It is also worth noting that the actual runtimes could be shorter than those reported in this study. For example, for direct and maternal genetic effects, as well as for direct and maternal SNP effects, of ssSNPBLUP_Liu, the Pearson correlations between estimates obtained when the termination criterion reached $10^{-5}$ (i.e. after 2032 iterations) and when it reached $10^{-6}$ (i.e. after 3358 iterations; Table 1) were all higher than 0.999. Further investigation on convergence criteria applied to ssSNPBLUP are therefore needed.

Our splitting of the coefficient matrix ${\mathbf {C}}$ of the two ssSNPBLUP systems into multiple parts to efficiently calculate its multiplication by a vector, should facilitate the implementation of ssSNPBLUP in breeding value estimation software currently used in animal breeding. Indeed, current software that implement ssGBLUP have already procedures to perform efficiently multiplications involving matrices such as ${\mathbf {C}}_{i_{LS}}$, ${\mathbf {C}}_{i_{R1}}$, or ${\mathbf {C}}_{i_{R2}}$ [16, 18]. To enable running ssSNPBLUP with those software, requires the implementation of at least two multiplications of the centered genotype matrix by an array. Finally, the computational strategies for two ssSNPBLUP systems proposed in this manuscript can be readily adapted for other ssSNPBLUP systems proposed in the literature [7, 8, 32].

## Conclusions

Based on the proposed approaches and our results, we showed that ssSNPBLUP provides a feasible approach to estimate genomic breeding values using current computers without resource to graphics processing units or special architecture. Using the Plink 1 binary form efficiently throughout the whole breeding value estimation process is relatively straightforward with a ssSNPBLUP approach, and allows to include more genotyped animals in a single-step evaluation than other single-step approaches with a same amount of RAM. The ssSNPBLUP approach proposed by Liu et al. [9] in combination with the Plink 1 binary form and solved with a PCG method with a second-level preconditioner was shown to be the most efficient approach in terms of memory and time requirements.

## Supplementary information

**Additional file 1.** Derivation of formula to approximate *diag*${\mathbf{Z^{\prime}A}}_{{gg}}^{{ - {\mathbf{1}}}} {\mathbf{Z}}$ and *diag*$(\bf{Z}'{{A}^{\it{gn}}}{{({{A}^{\it{nn}}})}^{-1}}{{A}^{\it{ng}}}Z)$ . Derivation of formula to approximate *diag*$\bf{{Z}'A}_{\it{gg}}^{-1}Z$and *diag*$(\bf{Z}'{{A}^{\it{gn}}}{{({{A}^{\it{nn}}})}^{-1}}{{A}^{\it{ng}}}Z)$[33–35].

**Additional file 2.**Description of the system of equations of ssSNPBLUP MS for a bivariate maternal model. Description of the system of equations of ssSNPBLUP MS for a bivariate maternal model, as well as of the different submatrices needed for the proposed computational strategies.

**Additional file 3.** Derivation of an alternative computation of $\bf{{A}^{\it{gn}}}{{({{A}^{\it{nn}}})}^{-1}}{{A}^{\it{ng}}}$Derivation of an alternative computation of $\bf{{A}^{\it{gn}}}{{({{A}^{\it{nn}}})}^{-1}}{{A}^{\it{ng}}}$that used only the ancestors and the progeny of the genotyped animals, instead of the complete pedigree.
