# Supplementary material for: Computational strategies for the preconditioned conjugate gradient method applied to ssSNPBLUP, with an application to a multivariate maternal model
Source: Genet Sel Evol. 2020 May 13;52:24. doi: 10.1186/s12711-020-00543-9 (PMC7222437; doi:10.1186/s12711-020-00543-9)
Supplement: Supplementary file 1 — Additional file 1. Derivation of formula to approximate diag${\mathbf{Z^{\prime}A}}_{{gg}}^{{ - {\mathbf{1}}}} {\mathbf{Z}}$Z′Agg-1Z and diag$(\bf{Z}'{{A}^{\it{gn}}}{{({{A}^{\it{nn}}})}^{-1}}{{A}^{\it{ng}}}Z)$(Z′Agn(Ann)-1AngZ) . Derivation of formula to approximate diag$\bf{{Z}'A}_{\it{gg}}^{-1}Z$Z′Agg-1Zand diag$(\bf{Z}'{{A}^{\it{gn}}}{{({{A}^{\it{nn}}})}^{-1}}{{A}^{\it{ng}}}Z)$(Z′Agn(Ann)-1AngZ)[33–35]. [file 12711_2020_543_MOESM1_ESM.pdf]

Additional file 1: Approximation of $diag\left(\mathbf{Z}'\mathbf{A}_{gg}^{-1}\mathbf{Z}\right)$ and $diag\left(\mathbf{Z}'\mathbf{A}^{gn}\left(\mathbf{A}^{nn}\right)^{-1}\mathbf{A}^{ng}\mathbf{Z}\right)$

Let $\mathbf{Z}$ be a matrix that contains the SNP genotypes (coded as 0 for one homozygous genotype, 1 for the heterozygous genotype, or 2 for the alternate homozygous genotype) centered by their observed means. The observed mean of the i-th SNP is equal to twice the allele frequency p_i (for the allele coded as 1) of that SNP.

Let $\mathbf{A}^{-1} = \begin{bmatrix} \mathbf{A}^{nn} & \mathbf{A}^{ng} \\ \mathbf{A}^{gn} & \mathbf{A}^{gg} \end{bmatrix}$ be the inverse of the pedigree relationship matrix partitioned between n_n ungenotyped animals (denoted as n) and n_g genotyped animals (denoted as g).

Below we derive formula to approximate $diag\left(\mathbf{Z}'\mathbf{A}_{gg}^{-1}\mathbf{Z}\right)$ and $diag\left(\mathbf{Z}'\mathbf{A}^{gn}\left(\mathbf{A}^{nn}\right)^{-1}\mathbf{A}^{ng}\mathbf{Z}\right)$ under some assumptions. Finally, we compare the approximations to the exact values based on the genotype dataset used in this manuscript.

Approximation of $diag\left(\mathbf{Z}'\mathbf{A}_{gg}^{-1}\mathbf{Z}\right)$

Let's assume that there is no inbreeding and that all genotyped animals are unrelated (i.e. $\mathbf{A}_{gg}^{-1} = \mathbf{I}$). If follows that:

$$diag\left(\mathbf{Z}'\mathbf{A}_{gg}^{-1}\mathbf{Z}\right) = diag\left(\mathbf{Z}'\mathbf{Z}\right)$$

The i-th diagonal element of $diag\left(\mathbf{Z}'\mathbf{A}_{gg}^{-1}\mathbf{Z}\right)$ can therefore be approximated as

$$\mathbf{z}'_i\mathbf{A}_{gg}^{-1}\mathbf{z}_i = \mathbf{z}'_i\mathbf{z}_i \approx 2p_i\left(1-p_i\right)n_g \tag{1}$$

where $\mathbf{z}_i$ is the i-th column of $\mathbf{Z}$, and $E\left(\mathbf{z}'_i\mathbf{z}_i\right) = 2p_i\left(1-p_i\right)n_g$ under the assumption of Hardy-Weinberg equilibrium [33].

To relax the assumption of no inbreeding and no relationships among genotyped animals, let's assume an average pedigree-based inbreeding coefficient $\bar{f}$ for the n_g genotyped animals, yielding an expected value for the diagonal elements of $\mathbf{A}_{gg}$ is equal to $1+\bar{f}$. If we assume random mating and ignore that the rate of inbreeding across generations within the data is likely different from

0, then the expected value for the off-diagonal elements of $\mathbf{A}_{gg}$ is equal to $2\bar{f}$, because the inbreeding coefficient is equal to the kinship, and thus half the additive genetic relationship, between the parents [34, 35].

Therefore, we can approximate $\mathbf{A}_{gg}$ as:

$$\mathbf{A}_{gg} \approx \mathbf{I}\left(1-\bar{f}\right) + \mathbf{2}\bar{f}\mathbf{2}'$$

where $\mathbf{2}$ is a column-vector with values equal to $\sqrt{2}$.

Then, using the Woodbury matrix identity, it follows that:

$$\begin{aligned}
\mathbf{A}_{gg}^{-1} &\approx \mathbf{I}\frac{1}{1-\bar{f}} - \mathbf{I}\frac{1}{1-\bar{f}}\mathbf{2}\left(\bar{f}^{-1} + \mathbf{2}'\mathbf{I}\mathbf{2}\frac{1}{1-\bar{f}}\right)^{-1}\mathbf{2}'\mathbf{I}\frac{1}{1-\bar{f}} \\
&\approx \frac{1}{1-\bar{f}}\left(\mathbf{I} - \mathbf{2}\left(\bar{f}^{-1} + \frac{2n_g}{1-\bar{f}}\right)^{-1}\mathbf{2}'\frac{1}{1-\bar{f}}\right) \\
&\approx \frac{1}{1-\bar{f}}\left(\mathbf{I} - \mathbf{2}\left(\frac{1-\bar{f}}{2n_g}\right)\mathbf{2}'\frac{1}{1-\bar{f}}\right) \\
&\approx \frac{1}{1-\bar{f}}\left(\mathbf{I} - \mathbf{1}\mathbf{1}'\frac{1}{n_g}\right) \qquad (2)
\end{aligned}$$

with $\left(\bar{f}^{-1} + \frac{2n_g}{1-\bar{f}}\right)^{-1} \approx \frac{1-\bar{f}}{2n_g}$ for large n_g.

Using Eq. 2, the i-th diagonal element of the product $\mathbf{Z}'\mathbf{A}_{gg}^{-1}\mathbf{Z}$ can be approximated as:

$$\begin{aligned}
\mathbf{z}'_i\mathbf{A}_{gg}^{-1}\mathbf{z}_i &\approx \mathbf{z}'_i\left(\frac{1}{(1-\bar{f})}\left(\mathbf{I} - \mathbf{1}\mathbf{1}'\frac{1}{n_g}\right)\right)\mathbf{z}_i \\
&\approx \frac{1}{(1-\bar{f})}\left(\mathbf{z}'_i\mathbf{z}_i - \frac{1}{n_g}\mathbf{z}'_i\mathbf{1}\mathbf{1}'\mathbf{z}_i\right) \\
&\approx \frac{1}{(1-\bar{f})}\left(\mathbf{z}'_i\mathbf{z}_i\right) \\
&\approx \frac{2}{(1-\bar{f})}p_i\left(1-p_i\right)n_g \qquad (3)
\end{aligned}$$

where $\mathbf{z}'_i\mathbf{1} = 0$.

It is worth noting that Eq. 3 is equal to Eq. 1 when $\bar{f} = 0$. In our implementation, we assumed no inbreeding and implemented the following approximation: $\mathbf{z}'_i\mathbf{A}_{gg}^{-1}\mathbf{z}_i \approx 2p_i\left(1-p_i\right)n_g$.

Approximation of $diag\left(\mathbf{Z}'\mathbf{A}^{gn}\left(\mathbf{A}^{nn}\right)^{-1}\mathbf{A}^{ng}\mathbf{Z}\right)$

The product $\mathbf{Z}'\mathbf{A}^{gn}\left(\mathbf{A}^{nn}\right)^{-1}\mathbf{A}^{ng}\mathbf{Z}$ can be written as:

$$\mathbf{Z}'\mathbf{A}^{gn}\left(\mathbf{A}^{nn}\right)^{-1}\mathbf{A}^{ng}\mathbf{Z} = \mathbf{Z}'\mathbf{A}^{gg}\mathbf{Z} - \mathbf{Z}'\mathbf{A}_{gg}^{-1}\mathbf{Z}$$

Assuming an average pedigree inbreeding coefficient $\bar{f}$, no missing parents, no mating among genotyped animals, no parent-offspring relationship between genotyped animals, it follows that the matrix $\mathbf{A}^{gg}$ is a diagonal matrix, and that the j-th diagonal element of $\mathbf{A}^{gg}$ is equal to, following Henderson's rules [22]:

$$\frac{2 + 0.5\bar{n}_{offspring}}{1 - \bar{f}} = \frac{0.5\left(4 + \bar{n}_{offspring}\right)}{1 - \bar{f}}$$

where $\bar{n}_{offspring}$ is the average number of offspring of a genotyped animal.

The i-th diagonal element of the product $\mathbf{Z}'\mathbf{A}^{gg}\mathbf{Z}$ can therefore be approximated as:

$$\begin{aligned}
\mathbf{z'}_i\mathbf{A}^{gg}\mathbf{z}_i &\approx \mathbf{z'}_i\mathbf{z}_i\left(0.5\left(4 + \bar{n}_{offspring}\right)\right)\frac{1}{1-\bar{f}} \\
&\approx 2p_i\left(1-p_i\right)n_g\left(0.5\left(\bar{n}_{offspring} + 4\right)\right)\frac{1}{1-\bar{f}} \\
&\approx \frac{1}{1-\bar{f}}p_i\left(1-p_i\right)n_g\left(\bar{n}_{offspring} + 4\right) \qquad (4)
\end{aligned}$$

Finally, from Eq. (3) and (4), it follows that i-th diagonal element of the product $\mathbf{Z}'\mathbf{A}^{gn}\left(\mathbf{A}^{nn}\right)^{-1}\mathbf{A}^{ng}\mathbf{Z}$ can be approximed as:

$$\begin{aligned}
\mathbf{z'}_i\mathbf{A}^{gn}\left(\mathbf{A}^{nn}\right)^{-1}\mathbf{A}^{ng}\mathbf{z}_i &= \mathbf{z'}_i\mathbf{A}^{gg}\mathbf{z}_i - \mathbf{z'}_i\mathbf{A}_{gg}^{-1}\mathbf{z}_i \\
&\approx \frac{1}{1-\bar{f}}p_i\left(1-p_i\right)n_g\left(\bar{n}_{offspring} + 4\right) - \frac{2}{1-\bar{f}}p_i\left(1-p_i\right)n_g \\
&\approx \frac{1}{1-\bar{f}}p_i\left(1-p_i\right)\left(\bar{n}_{offspring}n_g + 2n_g\right) \\
&\approx \frac{1}{1-\bar{f}}p_i\left(1-p_i\right)\left(n_{offspring} + 2n_g\right) \qquad (5)
\end{aligned}$$

where $n_{offspring}$ is the total number of offspring of all genotyped animals.

In our implementation, we assumed no inbreeding and implemented the following approximation:
$\mathbf{z'}_i\mathbf{A}^{gn}\left(\mathbf{A}^{nn}\right)^{-1}\mathbf{A}^{ng}\mathbf{z}_i \approx p_i\left(1-p_i\right)\left(n_{offspring} + 2n_g\right)$.

Illustration

Figures 1 and 2 illustrate the approximations of $\mathbf{z'}_i\mathbf{A}_{gg}^{-1}\mathbf{z}_i$ by $2p_i\left(1-p_i\right)n_g$ and of $\mathbf{z'}_i\mathbf{A}^{gn}\left(\mathbf{A}^{nn}\right)^{-1}\mathbf{A}^{ng}\mathbf{z}_i$ by $p_i\left(1-p_i\right)\left(n_{offspring} + 2n_g\right)$, respectively, based on the 260,591 genotypes including 37,995 segregating SNPs and used in this manuscript. The pedigree included 29,885,286 animals, and the total number of offspring for all genotyped animals was equal to 18,799,162. While it was not considered in this illustration and in our implementation, the average pedigree inbreeding coefficient for all genotyped animals was equal to 0.05.

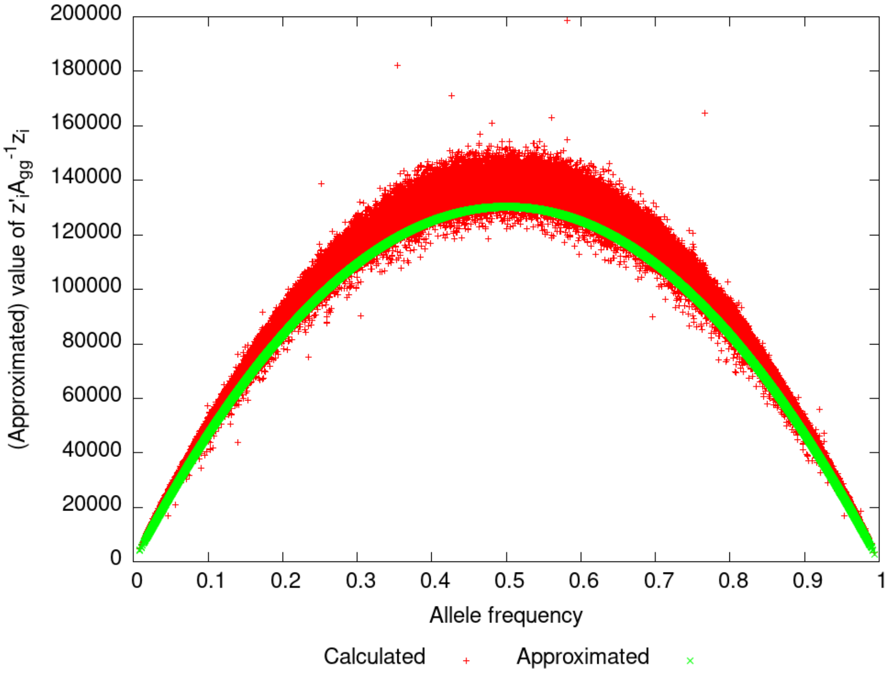

Figure 1: Calculated and approximated values of $diag\left(\mathbf{Z}'\mathbf{A}_{gg}^{-1}\mathbf{Z}\right)$

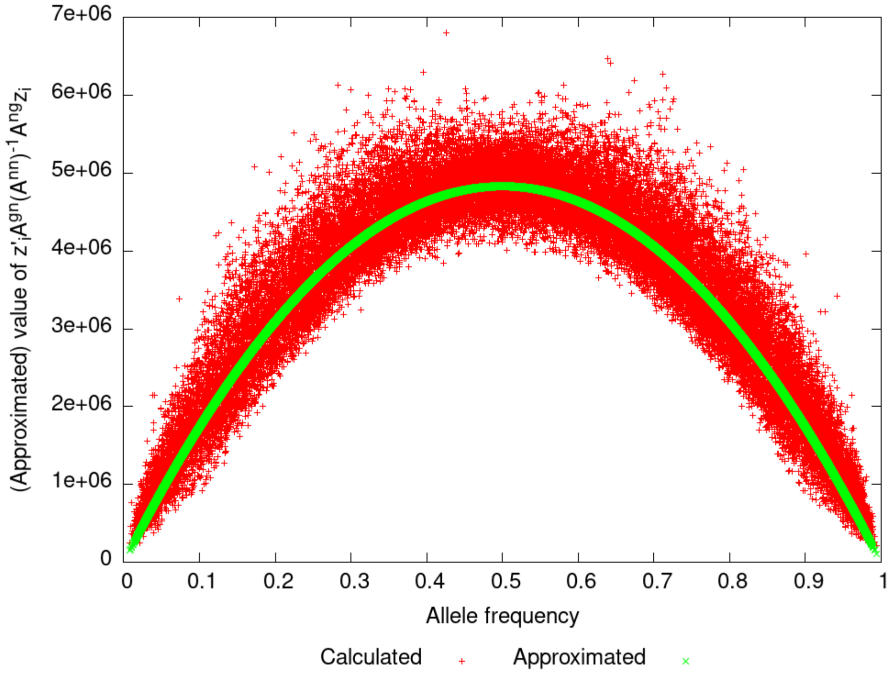

Figure 2: Calculated and approximated values of $diag\left(\mathbf{Z}'\mathbf{A}^{gn}\left(\mathbf{A}^{nn}\right)^{-1}\mathbf{A}^{ng}\mathbf{Z}\right)$
