# Supplementary material for: Computational strategies for the preconditioned conjugate gradient method applied to ssSNPBLUP, with an application to a multivariate maternal model
Source: Genet Sel Evol. 2020 May 13;52:24. doi: 10.1186/s12711-020-00543-9 (PMC7222437; doi:10.1186/s12711-020-00543-9)
Supplement: Supplementary file 2 — Additional file 2.Description of the system of equations of ssSNPBLUP MS for a bivariate maternal model. Description of the system of equations of ssSNPBLUP MS for a bivariate maternal model, as well as of the different submatrices needed for the proposed computational strategies. [file 12711_2020_543_MOESM2_ESM.pdf]

# Additional file 2: Description of the system of equations of ssSNPBLUP_MS for a bivariate maternal model

A standard bivariate maternal model associated with the ssSNPBLUP_MS system of equations can be written as (ignoring the permanent environmental effects):

$$\mathbf{y} = \mathbf{X}\boldsymbol{\beta} + \begin{bmatrix} \mathbf{W}_n & \mathbf{0} \\ \mathbf{0} & \mathbf{W}_g \end{bmatrix} \begin{bmatrix} \mathbf{u}_n \\ \mathbf{a}_g \end{bmatrix} + \begin{bmatrix} \mathbf{0} \\ \mathbf{W}_g\mathbf{M}_z \end{bmatrix} \mathbf{g} + \begin{bmatrix} \mathbf{S}_n & \mathbf{0} \\ \mathbf{0} & \mathbf{S}_g \end{bmatrix} \begin{bmatrix} \mathbf{m}_n \\ \mathbf{q}_g \end{bmatrix} + \begin{bmatrix} \mathbf{0} \\ \mathbf{S}_g\mathbf{M}_z \end{bmatrix} \mathbf{g}_m + \mathbf{e}$$

where the subscripts $g$ and $n$ refer to genotyped and non-genotyped animals, respectively, $\mathbf{y} = \begin{bmatrix} \mathbf{y}_{n,1} \\ \mathbf{y}_{n,2} \\ \mathbf{y}_{g,1} \\ \mathbf{y}_{g,2} \end{bmatrix}$ is the vector of records (with the subscripts 1 and 2 referring to the first and the second trait, respectively), $\boldsymbol{\beta} = \begin{bmatrix} \boldsymbol{\beta}_{n,1} \\ \boldsymbol{\beta}_{n,2} \\ \boldsymbol{\beta}_{g,1} \\ \boldsymbol{\beta}_{g,2} \end{bmatrix}$ is the vector of fixed effects, $\mathbf{u}_n = \begin{bmatrix} \mathbf{u}_{n,1} \\ \mathbf{u}_{n,2} \end{bmatrix}$ is the vector of direct genetic effects for non-genotyped animals, $\mathbf{a}_g = \begin{bmatrix} \mathbf{a}_{g,1} \\ \mathbf{a}_{g,2} \end{bmatrix}$ is the vector of direct residual polygenic effects for genotyped animals, $\mathbf{g} = \begin{bmatrix} \mathbf{g}_1 \\ \mathbf{g}_2 \end{bmatrix}$ is the vector of SNP effects for the direct genetic effects, $\mathbf{m}_n = \begin{bmatrix} \mathbf{m}_{n,1} \\ \mathbf{m}_{n,2} \end{bmatrix}$ is the vector of maternal genetic effects for non-genotyped animals, $\mathbf{q}_g = \begin{bmatrix} \mathbf{q}_{g,1} \\ \mathbf{q}_{g,2} \end{bmatrix}$ is the vector of maternal residual polygenic effects for genotyped animals, $\mathbf{g}_m = \begin{bmatrix} \mathbf{g}_{m,1} \\ \mathbf{g}_{m,2} \end{bmatrix}$ is the vector of SNP effects for the maternal genetic effects, and $\mathbf{e}$ is the vector of residuals.

The matrices $\mathbf{X} = \begin{bmatrix} \mathbf{X}_{n,1} & \mathbf{0} \\ \mathbf{0} & \mathbf{X}_{n,2} \\ \mathbf{X}_{g,1} & \mathbf{0} \\ \mathbf{0} & \mathbf{X}_{g,2} \end{bmatrix}$, $\mathbf{W}_n = \begin{bmatrix} \mathbf{W}_{n,1} & \mathbf{0} \\ \mathbf{0} & \mathbf{W}_{n,2} \end{bmatrix}$, $\mathbf{W}_g = \begin{bmatrix} \mathbf{W}_{g,1} & \mathbf{0} \\ \mathbf{0} & \mathbf{W}_{g,2} \end{bmatrix}$, $\mathbf{S}_n = \begin{bmatrix} \mathbf{S}_{n,1} & \mathbf{0} \\ \mathbf{0} & \mathbf{S}_{n,2} \end{bmatrix}$ and $\mathbf{S}_g = \begin{bmatrix} \mathbf{S}_{g,1} & \mathbf{0} \\ \mathbf{0} & \mathbf{S}_{g,2} \end{bmatrix}$ are incidence matrices relating records to their corresponding effects.
The matrix $\mathbf{M}_z$ is equal to $\mathbf{M}_z = \mathbf{I}_2 \otimes \mathbf{Z}$, with $\mathbf{I}_2$ being an identity matrix with size equal to the number of traits (i.e. 2) and the matrix $\mathbf{Z}$ containing the SNP genotypes (coded as 0 for one homozygous genotype, 1 for the heterozygous genotype, or 2 for the alternate homozygous genotype) centred by their observed means.

The system of linear equations for the bivariate ssSNPBLUP_MS can be written as follows:

$$\mathbf{Cx} = \mathbf{b}$$

where $\mathbf{C}$ is a symmetric positive (semi-)definite coefficient matrix, $\mathbf{x} = \begin{bmatrix} \hat{\boldsymbol{\beta}} \\ \hat{\mathbf{u}}_n \\ \hat{\mathbf{a}}_g \\ \hat{\mathbf{m}}_n \\ \hat{\mathbf{q}}_g \\ \hat{\mathbf{g}} \\ \hat{\mathbf{g}}_m \end{bmatrix}$ is the vector of solutions,

and $\mathbf{b} = \begin{bmatrix} \mathbf{X}'\mathbf{R}^{-1}\mathbf{y} \\ \mathbf{W}_n'\mathbf{R}_n^{-1}\mathbf{y}_n \\ \mathbf{W}_g'\mathbf{R}_g^{-1}\mathbf{y}_g \\ \mathbf{S}_n'\mathbf{R}_n^{-1}\mathbf{y}_n \\ \mathbf{S}_g'\mathbf{R}_g^{-1}\mathbf{y}_g \\ \mathbf{M}_z'\mathbf{W}_g'\mathbf{R}_g^{-1}\mathbf{y}_g \\ \mathbf{M}_z'\mathbf{S}_g'\mathbf{R}_g^{-1}\mathbf{y}_g \end{bmatrix}$ is the right-hand side with $\mathbf{R}^{-1} = \begin{bmatrix} \mathbf{R}_n^{-1} & \mathbf{0} \\ \mathbf{0} & \mathbf{R}_g^{-1} \end{bmatrix}$ being the inverse of the residual (co)variance structure matrix.

The matrix $\mathbf{C}$ is equal to $\mathbf{C} = \mathbf{C}_{LS} + \mathbf{C}_R$ with

$$\mathbf{C}_{LS} = \begin{bmatrix} \mathbf{X}'\mathbf{R}^{-1}\mathbf{X} & & & & & & \\ \mathbf{W}_n'\mathbf{R}_n^{-1}\mathbf{X}_n & \mathbf{W}_n'\mathbf{R}_n^{-1}\mathbf{W}_n & & & \mathit{symm.} & & \\ \mathbf{W}_g'\mathbf{R}_g^{-1}\mathbf{X}_g & \mathbf{W}_g'\mathbf{R}_n^{-1}\mathbf{W}_n & \mathbf{W}_g'\mathbf{R}_g^{-1}\mathbf{W}_g & & & & \\ \mathbf{S}_n'\mathbf{R}_n^{-1}\mathbf{X}_n & \mathbf{S}_n'\mathbf{R}_n^{-1}\mathbf{W}_n & \mathbf{S}_n'\mathbf{R}_n^{-1}\mathbf{W}_g & \mathbf{S}_n'\mathbf{R}_n^{-1}\mathbf{S}_n & & & \\ \mathbf{S}_g'\mathbf{R}_g^{-1}\mathbf{X}_g & \mathbf{S}_g'\mathbf{R}_n^{-1}\mathbf{W}_n & \mathbf{S}_g'\mathbf{R}_n^{-1}\mathbf{W}_g & \mathbf{S}_g'\mathbf{R}_n^{-1}\mathbf{S}_n & \mathbf{S}_g'\mathbf{R}_g^{-1}\mathbf{S}_g & & \\ \mathbf{M}_z'\mathbf{W}_g'\mathbf{R}_g^{-1}\mathbf{X}_g & \mathbf{M}_z'\mathbf{W}_g'\mathbf{R}_g^{-1}\mathbf{W}_n & \mathbf{M}_z'\mathbf{W}_g'\mathbf{R}_g^{-1}\mathbf{W}_g & \mathbf{M}_z'\mathbf{W}_g'\mathbf{R}_g^{-1}\mathbf{S}_n & \mathbf{M}_z'\mathbf{W}_g'\mathbf{R}_g^{-1}\mathbf{S}_g & \mathbf{M}_z'\mathbf{W}_g'\mathbf{R}_g^{-1}\mathbf{W}_g\mathbf{M}_z & \\ \mathbf{M}_z'\mathbf{S}_g'\mathbf{R}_g^{-1}\mathbf{X}_g & \mathbf{M}_z'\mathbf{S}_g'\mathbf{R}_g^{-1}\mathbf{W}_n & \mathbf{M}_z'\mathbf{S}_g'\mathbf{R}_g^{-1}\mathbf{W}_g & \mathbf{M}_z'\mathbf{S}_g'\mathbf{R}_g^{-1}\mathbf{S}_n & \mathbf{M}_z'\mathbf{S}_g'\mathbf{R}_g^{-1}\mathbf{S}_g & \mathbf{M}_z'\mathbf{S}_g'\mathbf{R}_g^{-1}\mathbf{W}_g\mathbf{M}_z & \mathbf{M}_z'\mathbf{S}_g'\mathbf{R}_g^{-1}\mathbf{S}_g\mathbf{M}_z \end{bmatrix}$$

and

$$\mathbf{C}_R = \begin{bmatrix} \mathbf{0} & & & & & & \\ \mathbf{0} & \mathbf{G}^{uu}\otimes\mathbf{A}^{nn} & & \mathit{symm.} & & & \\ \mathbf{0} & \mathbf{G}^{uu}\otimes\mathbf{A}^{gn} & \mathbf{G}^{uu}\otimes\boldsymbol{\Theta} & & & & \\ \mathbf{0} & \mathbf{G}^{mu}\otimes\mathbf{A}^{nn} & \mathbf{G}^{mu}\otimes\mathbf{A}^{ng} & \mathbf{G}^{mm}\otimes\mathbf{A}^{nn} & & & \\ \mathbf{0} & \mathbf{G}^{mu}\otimes\mathbf{A}^{gn} & \mathbf{G}^{mu}\otimes\boldsymbol{\Theta} & \mathbf{G}^{mm}\otimes\mathbf{A}^{gn} & \mathbf{G}^{mm}\otimes\boldsymbol{\Theta} & & \\ \mathbf{0} & \mathbf{G}^{uu}\otimes\mathbf{Z}'\mathbf{A}^{gn} & \mathbf{G}^{uu}\otimes\mathbf{Z}'\mathbf{Q} & \mathbf{G}^{um}\otimes\mathbf{Z}'\mathbf{A}^{gn} & \mathbf{G}^{um}\otimes\mathbf{Z}'\mathbf{Q} & \mathbf{G}^{uu}\otimes\left(\mathbf{Z}'\mathbf{QZ}+\frac{m}{1-w}\mathbf{I}\right) & \\ \mathbf{0} & \mathbf{G}^{mu}\otimes\mathbf{Z}'\mathbf{A}^{gn} & \mathbf{G}^{mu}\otimes\mathbf{Z}'\mathbf{Q} & \mathbf{G}^{mm}\otimes\mathbf{Z}'\mathbf{A}^{gn} & \mathbf{G}^{mm}\otimes\mathbf{Z}'\mathbf{Q} & \mathbf{G}^{mu}\otimes\left(\mathbf{Z}'\mathbf{QZ}+\frac{m}{1-w}\mathbf{I}\right) & \mathbf{G}^{mm}\otimes\left(\mathbf{Z}'\mathbf{QZ}+\frac{m}{1-w}\mathbf{I}\right) \end{bmatrix}$$

with $\boldsymbol{\Theta} = \frac{1}{w}\mathbf{A}^{gg} + \left(1 - \frac{1}{w}\right)\mathbf{Q}$ and with the inverse of the genetic (co)variance matrix between direct and maternal genetic effects being equal to $\mathbf{G}_0^{-1} = \begin{bmatrix} \mathbf{G}^{uu} & \mathbf{G}^{um} \\ \mathbf{G}^{mu} & \mathbf{G}^{mm} \end{bmatrix}$.

The matrix $\mathbf{Q}$ is equal to $\mathbf{Q} = \mathbf{A}^{gn}\left(\mathbf{A}^{nn}\right)^{-1}\mathbf{A}^{ng}$, with $\mathbf{A}^{-1} = \begin{bmatrix} \mathbf{A}^{nn} & \mathbf{A}^{ng} \\ \mathbf{A}^{gn} & \mathbf{A}^{gg} \end{bmatrix}$ being the inverse of the pedigree relationship matrix. The parameter $w$ is the proportion of variance (due to additive genetic effects) considered as residual polygenic effects and $m = 2\sum p_o\left(1 - p_o\right)$ with $p_o$ being the allele frequency of the $o^{th}$ SNP.

Following the Eq. (2) in the main text, the matrix $\mathbf{T}$ becomes $\mathbf{T} = \begin{bmatrix} \mathbf{I} & & & & & & \\ \mathbf{0} & \mathbf{I} & & & & & symm. \\ \mathbf{0} & \mathbf{0} & \mathbf{I} & & & & \\ \mathbf{0} & \mathbf{0} & \mathbf{0} & \mathbf{I} & & & \\ \mathbf{0} & \mathbf{0} & \mathbf{0} & \mathbf{0} & \mathbf{I} & & \\ \mathbf{0} & \mathbf{0} & \mathbf{0} & \mathbf{0} & \mathbf{0} & \mathbf{M}_z & \\ \mathbf{0} & \mathbf{0} & \mathbf{0} & \mathbf{0} & \mathbf{0} & \mathbf{0} & \mathbf{M}_z \end{bmatrix}$.

It follows that the matrix $\mathbf{C}_{MS_{LS}}$ in the Eq. (2) is equal to

$$\mathbf{C}_{MS_{LS}} = \begin{bmatrix} \mathbf{X}'\mathbf{R}^{-1}\mathbf{X} & & & & & & \\ \mathbf{W}_n'\mathbf{R}_n^{-1}\mathbf{X}_n & \mathbf{W}_n'\mathbf{R}_n^{-1}\mathbf{W}_n & & & & symm. & \\ \mathbf{W}_g'\mathbf{R}_g^{-1}\mathbf{X}_g & \mathbf{W}_g'\mathbf{R}_n^{-1}\mathbf{W}_n & \mathbf{W}_g'\mathbf{R}_g^{-1}\mathbf{W}_g & & & & \\ \mathbf{S}_n'\mathbf{R}_n^{-1}\mathbf{X}_n & \mathbf{S}_n'\mathbf{R}_n^{-1}\mathbf{W}_n & \mathbf{S}_n'\mathbf{R}_n^{-1}\mathbf{W}_g & \mathbf{S}_n'\mathbf{R}_n^{-1}\mathbf{S}_n & & & \\ \mathbf{S}_g'\mathbf{R}_g^{-1}\mathbf{X}_g & \mathbf{S}_g'\mathbf{R}_n^{-1}\mathbf{W}_n & \mathbf{S}_g'\mathbf{R}_n^{-1}\mathbf{W}_g & \mathbf{S}_g'\mathbf{R}_n^{-1}\mathbf{S}_n & \mathbf{S}_g'\mathbf{R}_g^{-1}\mathbf{S}_g & & \\ \mathbf{W}_g'\mathbf{R}_g^{-1}\mathbf{X}_g & \mathbf{W}_g'\mathbf{R}_g^{-1}\mathbf{W}_n & \mathbf{W}_g'\mathbf{R}_g^{-1}\mathbf{W}_g & \mathbf{W}_g'\mathbf{R}_g^{-1}\mathbf{S}_n & \mathbf{W}_g'\mathbf{R}_g^{-1}\mathbf{S}_g & \mathbf{W}_g'\mathbf{R}_g^{-1}\mathbf{W}_g & \\ \mathbf{S}_g'\mathbf{R}_g^{-1}\mathbf{X}_g & \mathbf{S}_g'\mathbf{R}_g^{-1}\mathbf{W}_n & \mathbf{S}_g'\mathbf{R}_g^{-1}\mathbf{W}_g & \mathbf{S}_g'\mathbf{R}_g^{-1}\mathbf{S}_n & \mathbf{S}_g'\mathbf{R}_g^{-1}\mathbf{S}_g & \mathbf{S}_g'\mathbf{R}_g^{-1}\mathbf{W}_g & \mathbf{S}_g'\mathbf{R}_g^{-1}\mathbf{S}_g \end{bmatrix},$$

that the matrix $\mathbf{C}_{MS_{R1}}$ in the Eq. (2) is equal to

$$\mathbf{C}_{MS_{R1}} = \begin{bmatrix} \mathbf{0} & & & & & & \\ \mathbf{0} & \mathbf{0} & & & symm. & & \\ \mathbf{0} & \mathbf{0} & \mathbf{G}^{uu} \otimes \left(\left(1-\frac{1}{w}\right)\mathbf{Q}\right) & & & & \\ \mathbf{0} & \mathbf{0} & \mathbf{0} & \mathbf{0} & & & \\ \mathbf{0} & \mathbf{0} & \mathbf{G}^{mu} \otimes \left(\left(1-\frac{1}{w}\right)\mathbf{Q}\right) & \mathbf{0} & \mathbf{G}^{mm} \otimes \left(\left(1-\frac{1}{w}\right)\mathbf{Q}\right) & & \\ \mathbf{0} & \mathbf{G}^{uu} \otimes \mathbf{A}^{gn} & \mathbf{G}^{uu} \otimes \mathbf{Q} & \mathbf{G}^{um} \otimes \mathbf{A}^{gn} & \mathbf{G}^{um} \otimes \mathbf{Q} & \mathbf{G}^{uu} \otimes \mathbf{Q} & \\ \mathbf{0} & \mathbf{G}^{mu} \otimes \mathbf{A}^{gn} & \mathbf{G}^{mu} \otimes \mathbf{Q} & \mathbf{G}^{mm} \otimes \mathbf{A}^{gn} & \mathbf{G}^{mm} \otimes \mathbf{Q} & \mathbf{G}^{mu} \otimes \mathbf{Q} & \mathbf{G}^{mm} \otimes \mathbf{Q} \end{bmatrix}$$

and that the matrix $\mathbf{C}_{MS_{R2}}$ in the Eq. (2) is equal to

$$\mathbf{C}_{MS_{R2}} = \begin{bmatrix} \mathbf{0} & & & & & & \\ \mathbf{0} & \mathbf{G}^{uu} \otimes \mathbf{A}^{nn} & & symm. & & & \\ \mathbf{0} & \mathbf{G}^{uu} \otimes \mathbf{A}^{gn} & \mathbf{G}^{uu} \otimes \frac{1}{w}\mathbf{A}^{gg} & & & & \\ \mathbf{0} & \mathbf{G}^{mu} \otimes \mathbf{A}^{nn} & \mathbf{G}^{mu} \otimes \mathbf{A}^{ng} & \mathbf{G}^{mm} \otimes \mathbf{A}^{nn} & & & \\ \mathbf{0} & \mathbf{G}^{mu} \otimes \mathbf{A}^{gn} & \mathbf{G}^{mu} \otimes \frac{1}{w}\mathbf{A}^{gg} & \mathbf{G}^{mm} \otimes \mathbf{A}^{gn} & \mathbf{G}^{mm} \otimes \frac{1}{w}\mathbf{A}^{gg} & & \\ \mathbf{0} & \mathbf{0} & \mathbf{0} & \mathbf{0} & \mathbf{0} & \mathbf{G}^{uu} \otimes \left(\frac{m}{1-w}\mathbf{I}\right) & \\ \mathbf{0} & \mathbf{0} & \mathbf{0} & \mathbf{0} & \mathbf{0} & \mathbf{G}^{mu} \otimes \left(\frac{m}{1-w}\mathbf{I}\right) & \mathbf{G}^{mm} \otimes \left(\frac{m}{1-w}\mathbf{I}\right) \end{bmatrix}$$

With the matrices $\mathbf{C}_{MS_{LS}}$, $\mathbf{C}_{MS_{R1}}$, and $\mathbf{C}_{MS_{R2}}$ being defined, the multiplication of the coefficient matrix $\mathbf{C}$, associated with a bivariate maternal ssSNPBLUP_MS, by a vector can be performed as proposed in the main text. For example, the multiplication of $\mathbf{C}_{MS_{LS}}$ by a vector can be performed with approaches already developed in animal breeding, such as iteration-on-data approaches [16-19]. Indeed, the block matrix $\begin{bmatrix} \mathbf{X}'\mathbf{R}^{-1}\mathbf{X} & & & & \\ \mathbf{W}_n'\mathbf{R}_n^{-1}\mathbf{X}_n & \mathbf{W}_n'\mathbf{R}_n^{-1}\mathbf{W}_n & & & symm. \\ \mathbf{W}_g'\mathbf{R}_g^{-1}\mathbf{X}_g & \mathbf{W}_g'\mathbf{R}_n^{-1}\mathbf{W}_n & \mathbf{W}_g'\mathbf{R}_g^{-1}\mathbf{W}_g & & \\ \mathbf{S}_n'\mathbf{R}_n^{-1}\mathbf{X}_n & \mathbf{S}_n'\mathbf{R}_n^{-1}\mathbf{W}_n & \mathbf{S}_n'\mathbf{R}_n^{-1}\mathbf{W}_g & \mathbf{S}_n'\mathbf{R}_n^{-1}\mathbf{S}_n & \\ \mathbf{S}_g'\mathbf{R}_g^{-1}\mathbf{X}_g & \mathbf{S}_g'\mathbf{R}_n^{-1}\mathbf{W}_n & \mathbf{S}_g'\mathbf{R}_n^{-1}\mathbf{W}_g & \mathbf{S}_g'\mathbf{R}_n^{-1}\mathbf{S}_n & \mathbf{S}_g'\mathbf{R}_g^{-1}\mathbf{S}_g \end{bmatrix}$ extracted from $\mathbf{C}_{MS_{LS}}$ is equal to the least-squares part of the coefficient matrix of a system of equations associated with a traditional pedigree BLUP based on the same model and datasets [19].
