# Supplementary material for: Computational strategies for the preconditioned conjugate gradient method applied to ssSNPBLUP, with an application to a multivariate maternal model
Source: Genet Sel Evol. 2020 May 13;52:24. doi: 10.1186/s12711-020-00543-9 (PMC7222437; doi:10.1186/s12711-020-00543-9)
Supplement: Supplementary file 3 — Additional file 3. Derivation of an alternative computation of $\bf{{A}^{\it{gn}}}{{({{A}^{\it{nn}}})}^{-1}}{{A}^{\it{ng}}}$Agn(Ann)-1AngDerivation of an alternative computation of $\bf{{A}^{\it{gn}}}{{({{A}^{\it{nn}}})}^{-1}}{{A}^{\it{ng}}}$Agn(Ann)-1Angthat used only the ancestors and the progeny of the genotyped animals, instead of the complete pedigree. [file 12711_2020_543_MOESM3_ESM.pdf]

Additional file 3: Derivation of an alternative computation of $\mathbf{A}^{gn}\left(\mathbf{A}^{nn}\right)^{-1}\mathbf{A}^{ng}$

Let the inverse of the pedigree relationship matrix of all animals in the pedigree, $\mathbf{A}^{-1}$, be partitioned between the genotyped animals g, and the non-genotyped animals n:
$\mathbf{A}^{-1}=\left[\begin{array}{cc}\mathbf{A}^{nn} & \mathbf{A}^{ng}\\ \mathbf{A}^{gn} & \mathbf{A}^{gg}\end{array}\right].$

Let the inverse of the pedigree relationship matrix of all genotyped animals and their ancestors, $\mathbf{A}_{anc}^{-1}$, be partitioned between the genotyped animals g, and the non-genotyped ancestors of the genotyped animals:
$\mathbf{A}_{anc}^{-1}=\left[\begin{array}{cc}\mathbf{A}_{anc,nn} & \mathbf{A}_{anc,ng}\\ \mathbf{A}_{anc,gn} & \mathbf{A}_{anc,gg}\end{array}\right]^{-1}=\left[\begin{array}{cc}\mathbf{A}_{anc}^{nn} & \mathbf{A}_{anc}^{ng}\\ \mathbf{A}_{anc}^{gn} & \mathbf{A}_{anc}^{gg}\end{array}\right].$
It is worth noting that a non-genotyped ancestor can be an offspring of a genotyped animal.

The inverse of the pedigree relationship matrix among genotyped animals g is then equal to:
$\mathbf{A}_{gg}^{-1}=\mathbf{A}^{gg}-\mathbf{A}^{gn}\left(\mathbf{A}^{nn}\right)^{-1}\mathbf{A}^{ng}=\mathbf{A}_{anc}^{gg}-\mathbf{A}_{anc}^{gn}\left(\mathbf{A}_{anc}^{nn}\right)^{-1}\mathbf{A}_{anc}^{ng}$

Therefore, it follows that:

$$\begin{aligned}\mathbf{Q} &= \mathbf{A}^{gn}\left(\mathbf{A}^{nn}\right)^{-1}\mathbf{A}^{ng}\\ &= \mathbf{A}_{anc}^{gn}\left(\mathbf{A}_{anc}^{nn}\right)^{-1}\mathbf{A}_{anc}^{ng}+\mathbf{A}^{gg}-\mathbf{A}_{anc}^{gg}\\ &= \mathbf{A}_{anc}^{gn}\left(\mathbf{A}_{anc}^{nn}\right)^{-1}\mathbf{A}_{anc}^{ng}+\mathbf{\Delta}\end{aligned}$$

From Henderson's rules to construct directly the inverse of a pedigree relationship matrix $\mathbf{A}^{-1}$ [22], adding the contribution of an animal to $\mathbf{A}^{-1}$ will modify at most for 9 entries of $\mathbf{A}^{-1}$: three diagonal elements of $\mathbf{A}^{-1}$ corresponding to the entries of the animal, its sire, and its dam, four off-diagonal elements of $\mathbf{A}^{-1}$ corresponding to the entries between the animal and its sire or its dam, and two off-diagonal elements of $\mathbf{A}^{-1}$ corresponding to the entries between its sire and its dam.

Therefore, only the genotyped animals and their non-genotyped offspring that are also ancestors of genotyped animals, contribute to $\mathbf{A}_{anc}^{gg}$, and only the genotyped animals and all their non-genotyped offspring contribute to $\mathbf{A}^{gg}$. It follows that the difference between $\mathbf{A}^{gg}$ and $\mathbf{A}_{anc}^{gg}$, $\mathbf{\Delta}$, is only due to the contributions to $\mathbf{A}^{-1}$ of the non-genotyped offspring of the genotyped animals that are not ancestors of genotyped animals. The matrix $\mathbf{\Delta}$ can be then easily

and directly constructed using Henderson's rules by reading the pedigree only once.
